# *Babesia*: An Emerging Infectious Threat in Transfusion Medicine

**DOI:** 10.1371/journal.ppat.1003387

**Published:** 2013-07-11

**Authors:** Cheryl A. Lobo, Jeny R. Cursino-Santos, Andy Alhassan, Marilis Rodrigues

**Affiliations:** Department of Blood-Borne Parasites, Lindsley Kimball Research Institute, New York Blood Center, New York, New York, United States of America; University of Wisconsin Medical School, United States of America

## What Is Babesiosis?

**Figure 1:**
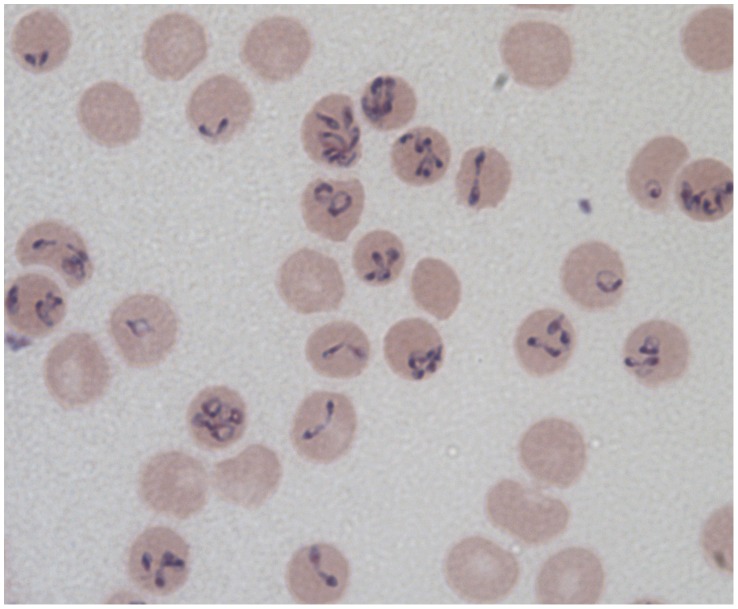
Different forms of *Babesia divergens* in human RBCs as seen on a Giemsa-stained smear from *in vitro* cultured parasites (ring, dividing figure eights, Maltese cross parasites, and multiply infected RBCs).

Babesiosis is an emerging zoonosis caused by protozoan parasites of the genus *Babesia*. The disease is endemic primarily in the Northeast and upper Midwestern United States. The genus *Babesia* comprises multiple species of apicomplexan parasites that infect red cells of many vertebrate hosts. *Babesia* divide and replicate in the hosts' red blood cells and are called piroplasms due to their pear-shaped appearance within the RBCs (Figure 1). They are transmitted by ixodid tick vectors as they feed on a blood meal from the host [1]. Babesiosis has long been recognized as an economically important disease of cattle, but only in the last 40 years has *Babesia* been recognized as an important pathogen in man.

The majority of cases in the United States are caused by *B. microti* and occur in the Northeast and upper Midwest [2]. A small number of infections caused by *B. duncani* and *B. duncani–*like organisms have been reported on the West Coast from California to Washington State [3]. Additionally, *B. divergens*–like organisms have been reported in Kentucky, Missouri, and Washington State [4]. In Europe, almost all reported cases have been due to *B. divergens*, and a few have been caused by the EU1 species, now called *B. venatorum* [5], [6]. Sporadic cases of babesiosis have also been reported in Asia, Africa, Australia, and South America [7].

The symptomatic spectrum of human babesiosis is broad, ranging from clinically silent infections to intense malaria-like episodes resulting occasionally in death. When present, symptoms typically are nonspecific (fever, headache, and myalgia) [8]. Human babesiosis is a zoonosis, and the natural acquisition of human disease is the result of interactions with established zoonotic cycles. Emerging diseases are defined as “novel, re-emerging, or drug-resistant infections whose incidence in humans has increased within the past two decades or whose incidence threatens to increase in the near future.” Because of the increasing number of human infections since 1990, human babesiosis can be considered an emerging disease [7]. A number of factors have contributed to the “emergence” of human babesiosis, including a heightened awareness among physicians, a changing ecology, and a larger population of immunocompromised individuals, where fatalities have occurred. This led the Centers for Disease Control and Prevention to add babesiosis to the list of nationally notifiable diseases in 2011.

## Why Has Babesiosis Become a Major Transfusion Threat?

In the United States, almost 5 million recipients undergo blood transfusions annually. These transfusion recipients are at potential risk of exposure to transmissible pathogens like *Babesia* from donor blood [9]. This is because, besides their natural route of transmission, the parasite is also transmitted by transfusion of blood products, as its red cell location provides an appropriate niche to facilitate its transmission. In fact, as the frequency of clinical cases has risen, there has been an associated increase in transfusion-transmitted *Babesia* (TTB) [10], making babesiosis the most frequent transfusion-transmitted infection with approximately 162 cases reported since 1980 and 12 associated fatalities in the period 2005–2008 [9], [10]. The major reason for this increase is that babesiosis can be asymptomatic, indeed clinically silent, in healthy adults who are the dominant blood donors. In one study, asymptomatic individuals who tested negative for *Babesia* in Giemsa smears had detectable amounts of *B. microti* DNA in their blood for three months [11]. Blood transfusion recipients generally present with more severe illness, as they have at least one of the risk factors for severe babesiosis, including extremes in age, lack of a spleen, hemoglobinopathies, cancers, HIV, and use of immunosuppressive therapy [9]. In these patients, babesiosis may be refractory to standard antimicrobial therapy [12] and may result in prolonged illness or death. Historically, babesiosis has been treated with a weekly course of clindamycin and quinine [13]. However, this combination of drugs can be so debilitating in some patients that it prevents successful completion of therapy. Physicians now recommend the equally effective combination of azithromycin and atovaquone [14]. Unfortunately, recent reports indicate that *B. microti* may become resistant to azithromycin-atovaquone in highly immunocompromised patients [12]. This drug resistance needs to be investigated further in the public health context. Among the 18 cases of TTB identified by the hemovigilance program at the American Red Cross between 2005 and 2007, ∼30% had a fatal outcome [15]. Some studies suggest a transmission risk as high as 1 per 601 blood units in areas of the highest prevalence [16]. To complicate this situation further, *B. microti* is known to survive and remain viable under blood storage conditions (4°C) for up to 35 days in RBCs and indefinitely in cryopreserved RBCs [17].

## What Is the Status of Current Blood-Banking Safeguards against Babesiosis?

The current strategy of blood screening, nationwide, to prevent transfusion-transmitted babesiosis (TTB) relies on a donor questionnaire to identify potential deferrals [10]. Donors who answer in the affirmative to a query of having a history of babesiosis are barred from donating from that day forward. This reliance on donor response to risk factor questions has many shortcomings as can be seen by the substantial increase in TTB in the last ten years. While it permanently excludes prospective blood donors with a history of babesiosis, it appears to be of limited value, presumably because infected blood donors experience asymptomatic infection or remain infectious long after symptoms have resolved. This current policy also impacts the blood supply because infectivity may be finite and patients who have had symptomatic babesiosis in the past might no longer be infectious. Systematic laboratory screening of the blood supply in the form of state-of-the-art FDA-licensed serological and nucleic acid testing (NAT) assays is available for many blood-borne pathogens like HIV and hepatitis to prevent their spread by transfusion. Unfortunately, the lack of comparable, sensitive screens available for vector-transmitted protozoal parasites like *Babesia* has resulted in the current complete dependence on a donor response questionnaire to safeguard the nation's blood supply.

## What Tests Are Currently Available to Detect *Babesia*?

The major barrier to preventing parasite transmission is the absence of a licensed assay to detect the parasite in blood donors. At the diagnostic level, there are both antibody and DNA detection tests available that are not suitable for blood donor screening for a number of reasons. The qPCR detection assays for *B. microti* exploit the gene encoding 18s rRNA as the template [18], [19] and have the advantage of detecting the parasite, if present, through the entire course of infection. As a diagnostic it is an efficient screen, as it is used to detect symptomatic infection where both parasite levels and volume of blood available for testing are high. Screening of donor blood for infectious organisms typically relies on a few milliliter sample of blood taken for analysis. As the parasite is present at a very low parasitemia in donor blood, this small sample of blood may not harbor sufficient parasites to yield a detectable amplification signal. This sampling limitation is currently the major impediment to deploying qPCR assays in donor blood testing. The antibody detection assay currently in use as a diagnostic is an immunofluorescence assay (IFA) screening for antibodies in sera of blood donors to smears of fixed parasites grown in hamster or mouse RBCs [18], [19]. Although rigorous data on the kinetics of the immune response to the parasite is not available, the window period in early infection, where antibodies are not present in circulation, may result in missed detection. Similarly, after the infection is resolved, antibodies may persist, resulting in erroneous positives that will exclude donors from giving blood. A high-throughput platform to enable use of IFA technology would also have to be developed to facilitate its use as a mass donor screen. Even when IFA and qPCR tests are combined, limitations persist, presenting an urgent need for a more sensitive and specific blood screening assay that can be used at blood centers for *Babesia* detection.

## Would Pathogen Reduction and/or Inactivation Technology Work for *Babesia*?

Pathogen reduction and inactivation technologies represent a different approach from testing donors for recognized pathogens. If sufficiently broad-spectrum and robust, they may prevent transmission of many emerging blood-borne infectious agents in the future, if they do not harm the recipient or blood component. Multiple pathogen inactivation (PI) technologies for the treatment of platelet or plasma components have been developed and are in routine use, based on methylene blue, psoralen, and riboflavin technologies. As *Babesia* is an intra-erythrocytic parasite, PI technologies would have to target the red cell component of the blood. No pathogen reduction technology for RBC units is commercially available at the present time, although there are two major platforms that are poised to change this status. The first is a technology (Cerus Corporation, Concord, California) based on the use of S-303, an intercalator group that inserts into the helical region of the nucleic acid, an effector group that allows covalent modification of nucleic acid, and a central frangible bond that allows degradation of the compound [20]. The second is the Mirasol PRT system (TerumoBCT Biotechnologies, Lakewood, Colorado), which uses a riboflavin additive that is UV light-activated to treat platelets, plasma, and whole blood [21]. Both processes have been shown to be effective against a variety of blood-borne pathogens, including *Babesia* species [22] (Cursino-Santos et al., unpublished data).

In summary, TTB has become one of the most commonly reported transfusion-transmitted infections in the United States. Thus, there is an urgent need to develop sensitive and specific methods for screening for this pathogen or alternative methods for eliminating it from the blood supply. Uncovering the basic biology of the parasite, including the identification of immuno-dominant antigens that could be used in designing more sensitive screens, may represent the path forward.
